# Regulation of CD4^+^ and CD8^+^ Effector Responses by Sprouty-1

**DOI:** 10.1371/journal.pone.0049801

**Published:** 2012-11-15

**Authors:** Sam Collins, Adam Waickman, Albert Basson, Abraham Kupfer, Jonathan D. Licht, Maureen R. Horton, Jonathan D. Powell

**Affiliations:** 1 Department of Medicine, Division of Pulmonary and Critical Care, Johns Hopkins University School of Medicine, Baltimore, Maryland, United States of America; 2 The Sidney Kimmel Comprehensive Cancer Center, Johns Hopkins University School of Medicine, Baltimore, Maryland, United States of America; 3 Department of Cell Biology, Johns Hopkins University School of Medicine, Baltimore, Maryland, United States of America; 4 Department of Craniofacial Development and Stem Cell Biology, Kings College, London, United Kingdom; 5 Division of Hematology/Oncology, Robert H. Lurie Comprehensive Cancer Center, Northwestern University Feinberg School of Medicine, Chicago, Illinois, United States of America; Saint Louis University School of Medicine, United States of America

## Abstract

TCR-induced NF-AT activation leads to the expression of both activating and inhibitory proteins. Previously, we had identified Egr-2 and Egr-3 as NF-AT-induced transcription factors which promote the inhibition of T cell activation. In this report we identify Sprouty1 as a downstream target of Egr-3. CD4^+^ T cells lacking Spry1 demonstrate enhanced proliferation and cytokine production. Likewise, Spry1^Flox/Flox^ Lck Cre CD8^+^ T cells display increased cytolytic activity. Mechanistically, Spry1 acts at the level of PLC-γ promoting the inhibition of both Ca^++^ induced NF-AT activation and MAP-kinase induced AP-1 activation while sparing NF-κB signaling. *In vivo*, mice in which Spry1 is selectively deleted in T cells demonstrate enhanced responses to a tumor vaccine and subsequently reject tumors more robustly than Wt mice. These findings suggest that targeting Spry1 might prove to be a novel means of enhancing tumor immunotherapy.

## Introduction

The selectivity of the TCR-MHC/peptide interaction confers the fine specificity of antigen recognition. TCR engagement in turn leads to robust downstream signaling resulting in the transcription of over 3,000 genes many of which are regulated in part by the canonical AP-1, NF-κB and NF-AT signaling pathways [1], [2]. In particular, NF-AT induced transcription in response to antigen recognition has been shown to play a critical role in the upregulation of both activating and inhibitory genes. Rao and colleagues have proposed that concomitant NF-AT and AP-1 activation, promoted in part by costimulation leads to the enrichment of the expression of genes associated with T cell activation [1]. Alternatively, NF-AT activation in the absence of AP-1 activation leads to the expression of a genetic program of inhibitory genes associated with T cell anergy. Indeed, the expression of the E3 ligases Cbl-b, ITCH and GRAIL and the kinase DGK-α that have been associated with negatively regulating T cell activation and promoting T cell anergy fit this paradigm [2], [3], [4], [5], [6], [7].

Previously, our lab identified the transcription factors Egr-2 and Egr-3 as being critical components of NF-AT induced negative regulation and anergy induction in Th1 cells [2], [8]. TCR engagement leads to the robust induction of both Egr-2 and Egr-3 in an NF-AT dependent manner. The expression of Egr-2 and Egr-3 is enhanced under conditions that promote anergy and their overexpression inhibits T-cell activation. Egr-3 null mice are relatively resistant to peptide induced anergy induction *in vivo*. Further, such mice are more susceptible in a mouse model of autoimmune pneumonitis. In addition, Egr-2 has been shown to promote the generation of IL-10 expressing Lag-3^+^ regulatory T cells [9]. Along these lines, Egr-2 null mice are more susceptible to developing lupus like autoimmune disorders [10].

We have proposed a model whereby TCR-induced expression of Egr-2 and Egr-3 promote a negative regulatory program by both inhibiting the induction of activating genes and enhancing the expression of inhibitory genes [8]. Indeed, the ability of the Egr-2 and Egr-3 to inhibit T cell function has been shown to be due to the direct inhibition of activating genes such as IL-2 as well as by promoting the expression of inhibitory genes such as Cbl-b. In an effort to better understand the ability of Egr-2 and Egr-3 to inhibit T cell function, we mined our previous microarray data set for potential Egr-target genes that might mediate this function [2]. In this report we identify a role for Sprouty-1 (Spry1) in regulating CD4^+^ and CD8^+^ T cell effector function. The Sprouty gene family consists of 4 proteins all of which have been shown to inhibit growth factor receptor signaling [11], [12]. Indeed, the genetic deletion of the various Sprouty genes leads to a wide variety of phenotypic abnormalities all associated with hyperactive receptor tyrosine kinase (RTK) signaling. Overall, the precise mechanisms by which the Spry proteins inhibit signaling is complex and diverse depending on the cell type. In this report we demonstrate the ability of Spry1 to regulate CD4^+^ and CD8^+^ T cell effector generation and function. Furthermore, we demonstrate that Spry1^Flox/Flox^ Lck Cre T cells acquire superior anti-tumor effector function. As such, our data identify Spry1 as a potentially novel target for enhancing tumor immunotherapy.

**Figure 1:**
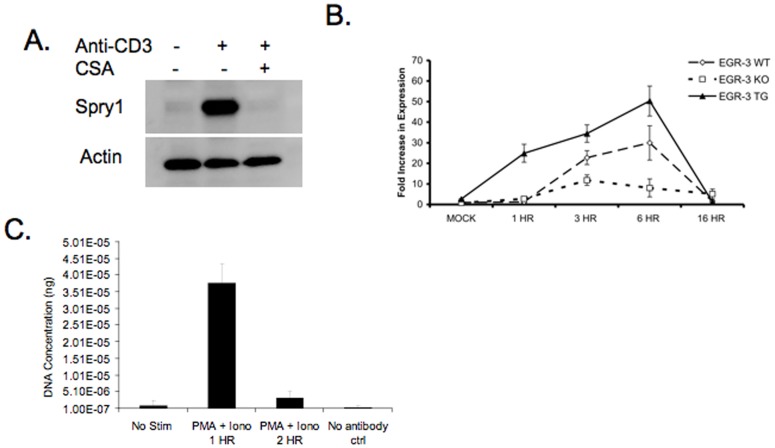
TCR-induced Spry1 is regulated by Egr-3. A. Western blot analysis of Spry1 and actin in previously activated 5C.C7 T cells stimulated with anti-CD3 with or without CSA treatment. **B.** Real-Time PCR analysis of Spry1 expression in wild type, Egr-3^−/−^ and Egr-3 overexpressing CD4^+^ T cells stimulated with anti-CD3 and anti-CD28. (N = 3). Mock treated cells were stimulated with anti-CD28 alone. **C.** CHiP analysis of Egr-3 binding to the Sprouty-1 promoter following PMA and Ionomycin stimulation. Error bars represent one standard deviation of the mean. All experiments were performed at least three times.

## Results

### TCR-induced Spry1 is Regulated by Egr-3

Previously, we had identified the transcription factors Egr-2 and Egr-3 as playing an important role in mediating the upregulation of genes associated with inhibition of T cell activation and promoting T cell anergy [2], [8]. TCR-induced Egr-2 and Egr-3 are dependent upon NF-AT activation and thus inhibited by cyclosporine A (CSA). Therefore, we mined microarray data for genes whose expression was inhibited by CSA and upregulated in T cells rendered anergic *in vivo* (data not shown). One of the genes identified was Spry1. We sought to reproduce the microarray data by stimulating cells with anti-CD3 in the presence and absence of CSA. Indeed, western blot analysis confirmed that Spry1 protein was upregulated by TCR engagement in the absence of costimulation and inhibited by CSA (Figure 1A). We had previously shown that Cbl-b, an inhibitor of T cell signaling and promoter of T cell anergy was regulated by Egr-3. Thus we wanted to determine whether the expression of Spry1 was due to the direct affects of NF-AT or the result of NF-AT-induced Egr-3 expression. T cells from Wt mice, those that lack Egr-3 and T cells from mice which overexpress Egr-3 were fully activated with anti-CD3+ anti-CD28 overnight and Spry1 expression was analyzed by RT-PCR. Wt T cells demonstrated increased expression of Spry1 upon activation (Figure 1B). T cells lacking Egr-3 displayed markedly decreased Spry1 expression upon TCR engagement while T cells engineered to overexpress Egr-3 had markedly increased expression of Spry1. These findings are consistent with the hypothesis that Spry1 is regulated by Egr-3, and that Spry1 expression is part of the Egr-3-mediated negative feedback loop. Spry1 has several Egr-3 binding sites and thus we wanted to directly demonstrate the ability of Egr-3 to bind to the Spry1 promoter. To this end T cells were activated with PMA and ionomycin and ChIP assays were performed. Chemical activation with PMA and ionomycin promotes the rapid and simultaneous activation of the T cells thus enhancing the robustness of the ChIP assay. Consistent with our findings employing Egr-3 null and overexpressing T cells, our ChIP experiments revealed that upon activation, Egr-3 binds to the Spry1 promoter (Figure 1C).

**Figure 2:**
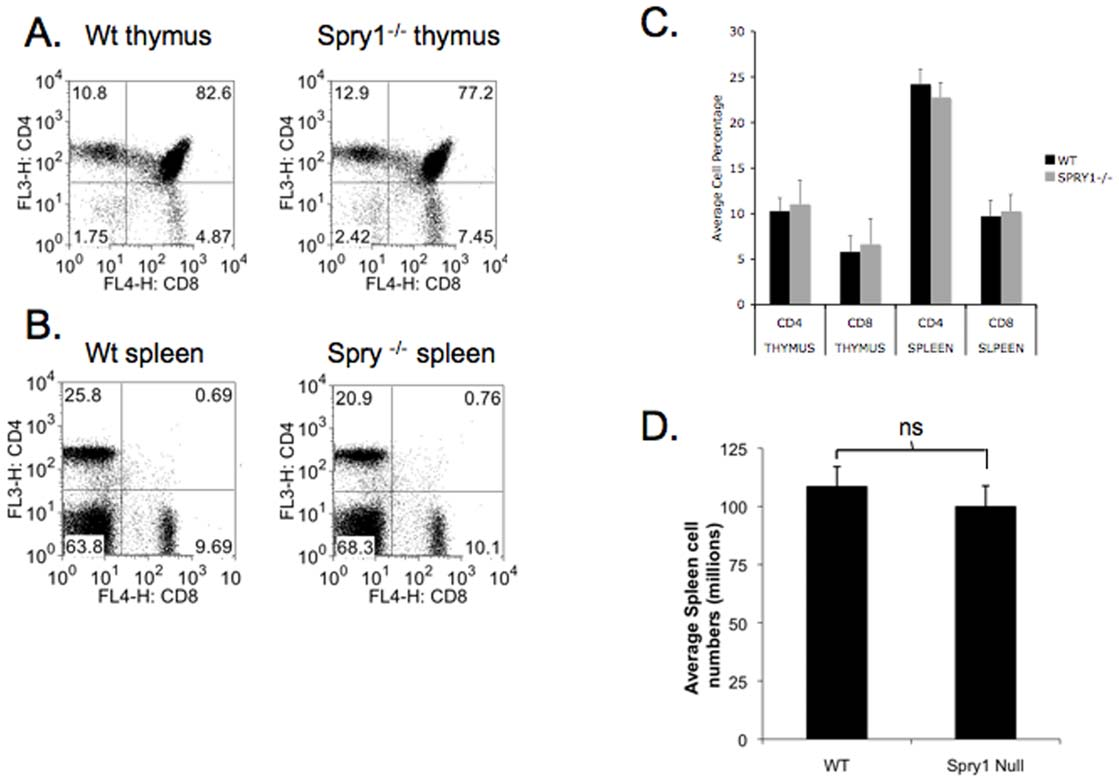
Loss of Spry1 in CD4^+^ and CD8^+^ T cells does not affect T cell development. B6 Lck Cre mice were crossed to Spry1 flox mice and thymuses (**A**) and spleens (**B**) were analyzed for CD4 and CD8 percentages by flow cytometry. **C.** Grapical representation of thymus and spleen CD4^+^ and CD8^+^ T cell percentages from wild type and Spry1^Flox/Flox^ Lck cre mice. **D.** Graphical representation of splenic cell numbers of wild type and Spry1^Flox/Flox^ Lck cre mice. Error bars represent one standard deviation of the mean. All experiments were performed at least three times. Data are representative of 5 mice per group.

### Spry1 Negatively Regulates CD4^+^ and CD8^+^ T cell Effector Function

Having identified Spry1 as a TCR-induced, Egr-3-dependent gene, we next wanted to determine the role of Spry1 in regulating T cell effector function. To accomplish this goal, we crossed mice in which the Spry1 gene had been flanked with LoxP sites with a mouse that was engineered to express cre recombinase under control of the Lck promoter. Mice homozygous for Spry1 Flox and Lck cre were generated and lymphoid organs analyzed for proper T cell development (Figure 2). Thymuses of Spry1^Flox/Flox^ Lck Cre mice contained CD4^+^, CD8^+^, and CD4^+^/CD8^+^ T cells at percentages that did not differ significantly from wild type (Figure 2A). Spleens of Spry1^Flox/Flox^ Lck Cre mice also appeared to have normal ratios of CD4^+^ and CD8^+^ T cells when compared to wild type mice (Figure 2B & C). In addition, total spleen cell numbers were not different between wild type and Spry1^Flox/Flox^ Lck Cre (Figure 2D). Overall, loss of Spry1 expression in T cells did not result in significant changes in cellular development or peripheral physiology of lymphoid organs.

**Figure 3:**
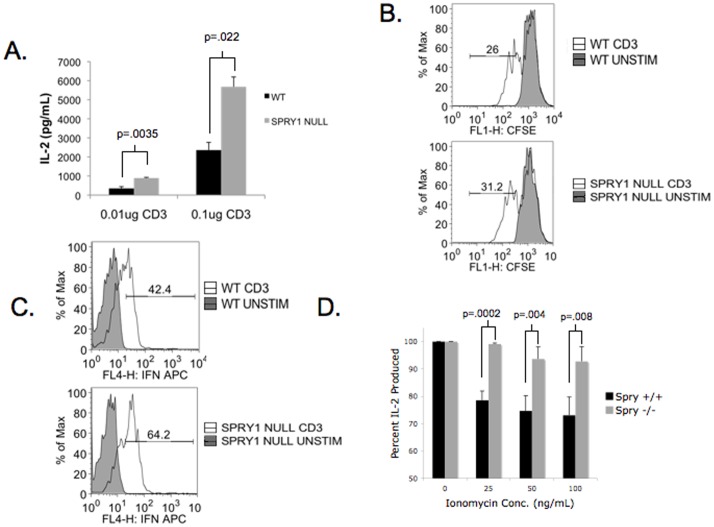
Spry1 null CD4^+^ T cells have enhanced effector function. Spry1 null and wild type spleens were expanded *in vitro* with anti-CD3; CD4^+^ T cells were isolated and rechallenged with increasing doses of anti-CD3. **A.** IL-2 production by CD4^+^ T cells was determined by ELISA. **B.** CFSE dilution of CD4^+^ wild type and Spry1 null CD4^+^ T cells. **C.** IFNγ production was determined by intracellular cytokine secretion assay. % of Max indicates the fraction of the total cells at a given fluorescent intensity. **D.** Following expansion Spry1 null and wild type spleens were stimulated with increasing doses of ionomycin, briefly rested and rechallenged with anti-CD3. Resistance to ionomycin-induced anergy is displayed as percentage of IL-2 produced following ionomycin treatment and rechallenge compared to rechallenged alone. Error bars represent one standard deviation of the mean. P values indicate statistical significance by student t-test. All experiments were performed at least three times. Data are representative of 3 mice per group.

First we wanted to determine the role of Spry1 in regulating CD4^+^ T cell function. To this end, we expanded wild type and Spry1^Flox/Flox^ Lck Cre spleens with anti-CD3 and IL-2. After seven days, CD4^+^ T cells were purified by MACS isolation and rechallenged with anti-CD28 and increasing concentrations of anti-CD3 for 24 hours. T cell function was determined by assaying cell supernatants for IL-2 production (Figure 3A). Spry1^Flox/Flox^ Lck Cre CD4^+^ T cells produced approximately two to three fold more IL-2 than wild type CD4^+^ T. Interestingly, this increase in IL-2 production did not result in a marked increase in proliferation as determined by CFSE dilution (Figure 3B). These observations suggest that with regard to proliferation, IL-2 production by Wt mice is not limiting and Spry1 does not appear to directly impact cell division. Of note CD25 levels in Wt and Spry1 null T cells were equivalent (data not shown). On the other hand, the expanded Spry1^Flox/Flox^ Lck Cre T cells produced 50% more IFN-γ upon rechallenge when compared to Wt T cells (Figure 3C). CD4^+^ T cell anergy was initially described as a block in Ras MAPK signaling. Since Spry1 has been shown to interact with multiple signaling molecules involved in Ras MAPK signaling, we wanted to determine the consequence of Spry1 deletion on the induction of CD4^+^ T cell anergy. To address this question, we utilized an ionomycin-induced anergy model where previously activated CD4^+^ T cells are stimulated with increasing concentrations of ionomycin overnight, briefly rested, and rechallenged with anti-CD3 and anti-CD28 (Figure 3D). Wild type T cells displayed susceptibility to ionomycin-induced anergy, represented by a drop in IL-2 production when treated with the highest dose of ionomycin. In contrast, Spry1^Flox/Flox^ Lck Cre CD4^+^ T cells were resistant to ionomycin-induced anergy, even at the highest dose of ionomycin. These data indicate that loss of Spry1 results in both enhanced CD4^+^ T cell function and resistance to ionomycin-induced anergy.

**Figure 4:**
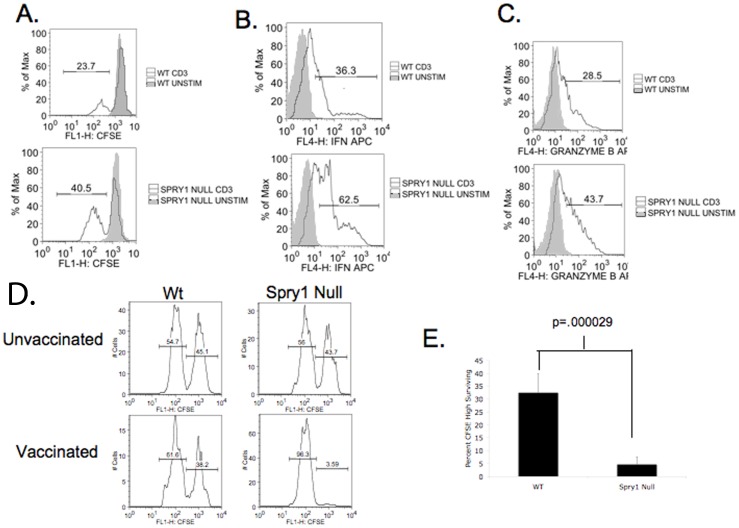
Spry1 null CD8^+^ T cells have enhanced effector function. Spry1 null and wild type spleens were expanded *in vitro* with anti-CD3, spleens were rechallenged with anti-CD3. **A.** CFSE dilution of wild type and Spry1 null CD8^+^ T cells. Intracellular cytokine secretion assay was performed for IFNγ (**B**) and Granzyme-B (**C**). A mixture of CFSE high labeled, peptide pulsed splenocytes and CFSE low unlabeled splenocytes were injected I. V. into either wild type or Spry1^Flox/Flox^ Lck Cre mice. Sixteen hours later splenocytes were analyzed by flow cytometry for the presence of CFSE high and CFSE low cells. **D.** Representative histogram data (for individual mice) from unvaccinated and vaccinated Wt and Spry1^Flox/Flox^ Lck Cre mice. Percentages are representative of CFSE high and CFSE low populations. **E.** Graphical representation of data from five wild type and five Spry1^Flox/Flox^ Lck Cre mice. Error bars represent one standard deviation of the mean. P values indicate statistical significance by student t-test. All experiments were performed at least three times.

**Figure 5:**
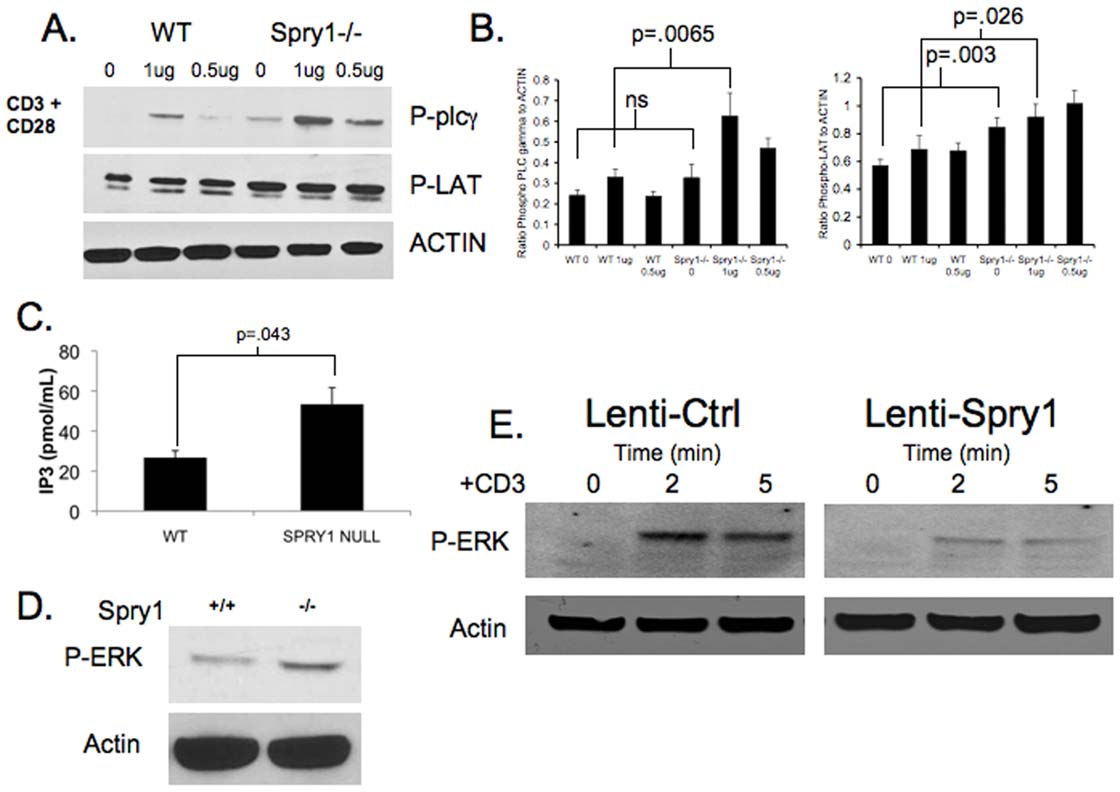
Spry1 null CD4^+^ T cells have enhanced TCR signaling. **A.** Western blot analysis of PLC-γ and LAT phosphorylation in wild type and Spry1^flox/flox^ Lck cre CD4^+^ T cells following ten-minute stimulation with anti-CD3 and anti-CD28. **B.** Graphical representation of Phospho-PLCγ and Phospho-LAT levels as measured by densitometry presented as ratio of signal to Actin levels. **C**. IP3 levels in wild type and Spry1 null CD4^+^ T cells following five-minute stimulation with anti-CD3. **D.** Western blot analysis of ERK phosphorylation in wild type and Spry1^flox/flox^ Lck cre CD4^+^ T cells following 5 minute stimulation with anti-CD3. **E.** Western blot analysis of ERK phosphorylation in control vector or Spry1 overexpression vector transduced jurkat T cells following 2 and 5 minute stimulation with anti-CD3. Error bars represent one standard deviation of the mean. P values indicate statistical significance by student t-test. All experiments were performed at least three times. Data are representative of 3 mice per group.

Next we sought to determine the role of Spry1 in regulating CD8^+^ T cell effector function. Wt and Spry1^Flox/Flox^ Lck Cre spleens were expanded and previously activated CD8^+^ T cells were isolated. Interestingly, as measured by CFSE dilution, Spry1^Flox/Flox^ Lck Cre CD8^+^ T cells demonstrated increased proliferation when compared to Wt cells (Figure 4A). At this time it is not precisely clear why proliferation of CD8+ T cells was increased in the Spry1 null T cells but not in the CD4+ T cells. Nonetheless similar to CD4+ T cells, Spry1^Flox/Flox^ Lck Cre CD8^+^ T cells also had increased percentages of IFN^+^ producing cells, (wt 36.3%: Spry1 62.5%) and Granzyme-B^+^ cells (wt 28.5%: null 43.7%) (Figure 4B & C). These results demonstrate the ability of Spry1 to negatively regulate effector cytokine production in both CD4+ and CD8+ T cells. We next wanted to determine the role of Spry1 in regulating the ability of CD8^+^ effector cells to kill. To accomplish this, we utilized an *in vivo* cytotoxic T lymphocyte (CTL) assay. Wild type and Spry1^Flox/Flox^ Lck Cre mice were given an I.P. injection of a recombinant vaccinia virus expressing ovalbumin. One week later, vaccinated mice were given an I.V. injection of a 1∶1 mixture of CFSE hi stained syngeneic splenocytes pulsed with OVA peptide and unpulsed CFSE low stained splenocytes. Sixteen hours later, spleens were isolated from the previously vaccinated mice, processed to single cell suspensions, and analyzed by flow cytometry to determine the ratio of CFSE low to CFSE high cells remaining in the recipient mice (Figure 4D,E). Overall, the Spry1^Flox/Flox^ Lck Cre T cell mice demonstrated markedly enhanced antigen specific killing of the target cells when compared to the vaccinated wt mice.

**Figure 6:**
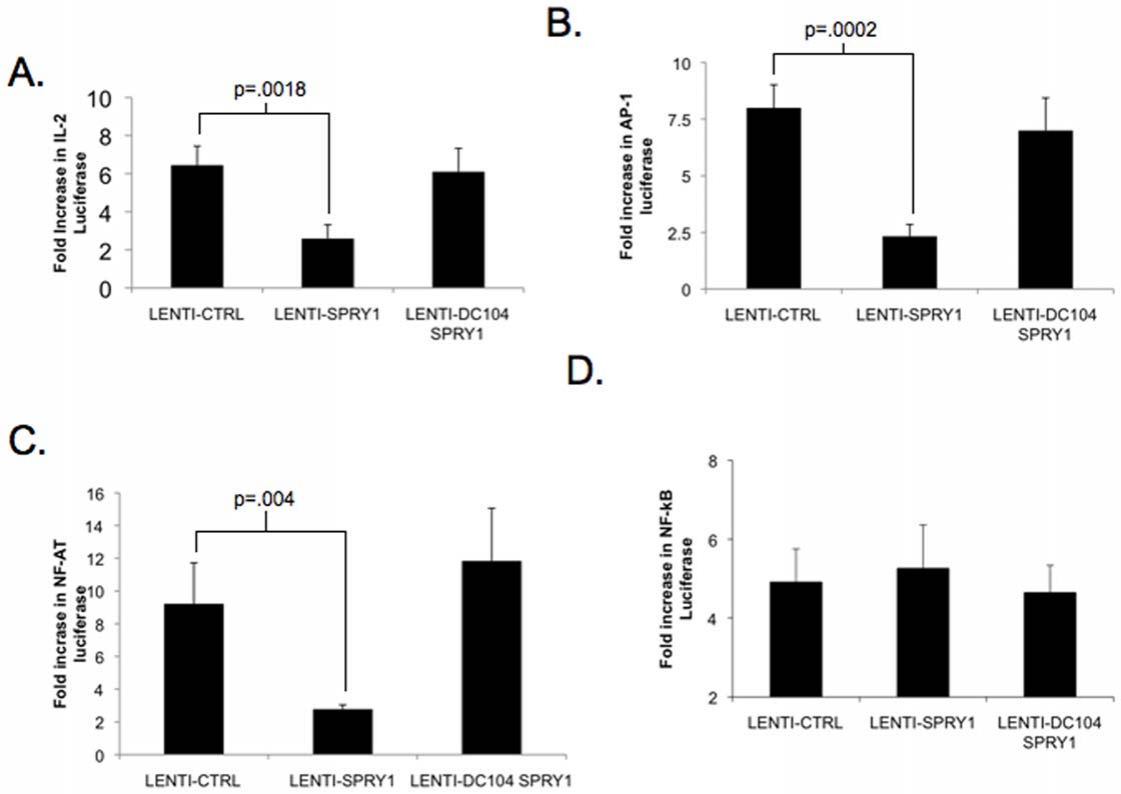
Spry1 overexpression suppresses IL-2, AP-1, and NF-AT signaling. Jurkat T cells were transfected with either control vector (Lenti-CTRL), Sprouty-1 overexpression vector (Lenti-Spry1) or a mutant Spry1 overexpression vector that lacks the c terminal domain (Lenti-DC104 Spry1). Jurkats were also tranfected with either an IL-2 driving luciferase construct (**A**), an AP-1 driving luciferase construct (**B**), an NF-AT driving luciferase construct(**C**), or an NF-κB driving luciferase construct (**D**). Jurkats were stimulated with anti-CD3 and luciferase activity was measured. Data are presented as fold increase in luciferase over unstimulated. Error bars represent one standard deviation of the mean. P values indicate statistical significance by student t-test. All experiments were performed at least three times.

### Spry1 Regulates TCR-induced AP-1 and NF-AT Activation in T cells

Next, we sought to determine the mechanism accounting for the enhanced function in Spry1 null T cells. Previously, it had been reported that over-expression of Spry1 in Jurkat T cells resulted in reduced LAT phosphorylation [13]. Therefore, we examined LAT phosphorylation in the absence of Spry1. Wild type and Spry1^Flox/Flox^ Lck Cre splenocytes were activated and expanded *in vitro* with anti-CD3 and IL-2 for 7 days. CD4^+^ T cells were subsequently isolated and stimulated with anti-CD28 and increasing concentrations of anti-CD3 for 5 minutes. Cell lysates were prepared, subjected to western blot, and probed with anti-phospho-LAT (Figure 5A & B). Spry1^Flox/Flox^ Lck Cre T cells demonstrated enhanced LAT phosphorylation when compared to Wt T cells. Furthermore, this enhanced activation corresponded to an even greater increase in downstream phosphorylation of PLC-γ.

**Figure 7:**
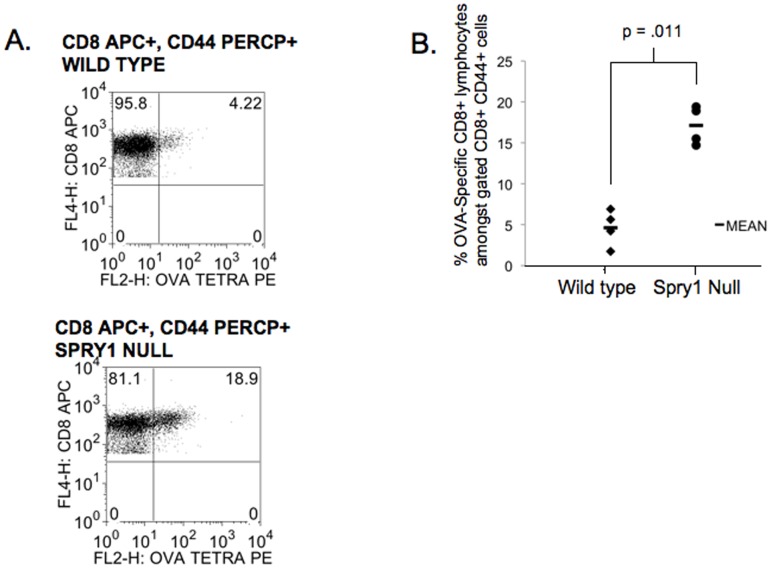
Spry1 null mice have increased percentages of OVA specific CD8^+^ T cells following vaccination. Wild type and Spry1 null mice were subcutaneously injected with a 1∶1 mixture of irradiated EL-4 tumor cells expressing ovalbumin and B16 cells expressing GM-CSF. Seven days later inguinal lymph nodes were isolated and stained with CD8 and ovalbumin specific tetramer. (**A**) Flow cytometric data from lymph nodes of one wild type and one Spry1 null mouse. Gates are set on CD8^+^ and CD44^+^ cells. (**B**) Graphical representation of data from four wild type and four Spry1 null mice. P values indicate statistical significance by student t-test. All experiments were performed at least three times.

Activation of PLC-γ leads to the generation of IP3. Therefore, we chose to analyze whether loss of Spry1 had any effect on IP3 levels. Spry1^Flox/Flox^ Lck Cre CD4^+^ T cells had IP3 levels that were approximately 2 fold greater than wild type CD4^+^ T cells (55.3 pmol: 27.6 pmol) (Figure 5C). In addition to IP3, PLC-γ activation leads to the generation of DAG. DAG in turn can promote the activation of the Ras MAP-kinase pathway. Consistent with the increased activation of PLC-γ, ERK phosphorylation was increased in Spry1^Flox/Flox^ Lck Cre T cells upon activation when compared to Wt T cells (Figure 5D). In order to confirm this finding, we transfected Jurkat T cells with either a control lenti-viral vector, or a lenti-viral vector with an over-expressed Spry1 construct. Transfected cells were sorted, stimulated with anti-CD3 for 2 and 5 minutes, and then examined for phospho-ERK by western blot analysis (Figure 5E). Consistent with the observation that T cells lacking Spry1 display enhanced ERK phosphorylation upon activation, overexpression of Spry1 leads to inhibition of ERK phosphorylation in T cells.

**Figure 8:**
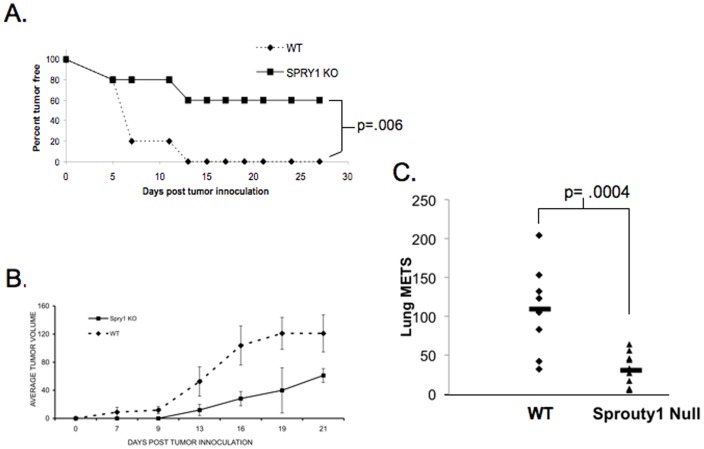
Spry1 null mice have enhanced clearance of EL-4 tumor cells. Wild type and Spry1 null mice were subcutaneously injected with EL-4 tumor cells. Mice were monitored for (**A**) tumor free survival and (**B**) tumor volume. All experiments were performed at least three times. (**C**) Wt and Spry1 null T cell mice were injected IV with 250,000 B16 melanoma cells, 14 days later mice were sacrificed and lung tumors were counted. P values indicate statistical significance by student t-test. All experiments were performed at least three times. Data are representative of 10 mice per group.

Previously, it had been shown that Spry1 could be recruited to the immunologic synapse upon activation, suggesting that Spry1 might be localized in central supramolecular clusters [13]. Since the Spry1^Flox/Flox^ Lck Cre T cells demonstrate increased LAT activation, which is also recruited to the synapse, we wanted to determine if such was the case in our system. A population of naïve (5C.C7 TCR transgenic T cells on a Rag^−/−^ background) were incubated with APC and cognate peptide for 20 minutes and then analyzed for the cellular localization of Spry1. Consistent with previously published findings, upon activation, Spry1 translocates to the synapse thus providing in part a mechanism whereby it can interfere with LAT activation (Figure S1).

Our signaling data demonstrate the ability of Spry1 to negatively regulate both the Ca^++^ and Ras-MAP-kinase pathways downstream of TCR signaling. Based on these findings, we hypothesized that Spry1 would affect the activation of the downstream transcription factors AP-1 (which is activated by the MAP-kinase pathway) and NF-AT (which is activated by the Ca^++^ pathway). To test this hypothesis we analyzed the effect of Spry1 over-expression on IL-2 promoter signaling, as well as NFAT, AP-1 and NF-κB induced transcription. Jurkat T cells were transfected with a control lenti-viral construct or a Spry1 expressing lenti-viral construct. In addition, we created a mutated Spry1 over-expression construct that lacks the c terminal cysteine rich domain (Figure S2). This domain has been implicated in directing Sprouty localization, and in light of our microscopy data (Figure S1) we sought to determine if Spry1 translocation was necessary for Spry1 function. As a readout, the cells were also transfected with various promoter driven luciferase reporter constructs. The cells were stimulated with anti-CD3 overnight and luciferase activity was measured (Figure 6). Consistent with our observation that Spry1 null T cells produce more IL-2 than Wt T cells, the overexpression of Spry1 led to inhibition of IL-2 promoter driven luciferase activity (Figure 6A). Furthermore, Spry1 overexpression specifically inhibited the expression of AP-1 and NF-AT driven reporter activity. These observations are consistent with the ability of Spry1 to inhibit TCR-induced Ca^++^ and MAP-kinase signaling (Figure 6B & C). Alternatively, Spry1 overexpression did not inhibit TCR-induced activation of NF-κB driven reported activity (Figure 6D). Interestingly, elimination of the cysteine rich c terminal domain resulted in complete abrogation of suppression of IL-2, NF-AT and AP-1 induced luciferase transcription. Of note, this lack of inhibition by the mutated Spry1 construct was not secondary to decreased expression (Figure S3). These observations support our findings that the ability of Spry1 to inhibit signaling is mechanistically facilitated by its translocation to the cSMAC.

### Spry1 Null T cells Demonstrate Enhanced Anti-tumor Activity

Thus far our data support a role for Spry1 in mediating inhibition of T cell effector function. Therefore we hypothesized that Spry1^Flox/Flox^ Lck Cre T cells would demonstrate enhanced anti-tumor activity. To test this hypothesis we first tested the ability of Spry1^Flox/Flox^ Lck Cre T cells to become activated in response to the whole cell anti-tumor vaccine GVAX [14]. Wild type and Spry1^Flox/Flox^ Lck Cre mice were subcutaneously injected with a 1∶1 mixture of irradiated ovalbumin expressing EL-4 cells and irradiated GM-CSF secreting B16 cells. Seven days later, inguinal lymph nodes were isolated and analyzed by flow cytometry for ovalbumin specific CD8^+^ T cells by MHC class I tetramer staining (Figure 7). Lymph nodes from Spry1^Flox/Flox^ Lck Cre mice had 3 times as many ovalbumin specific CD8^+^ T cells as wild type mice. These data indicate that Spry1^Flox/Flox^ Lck Cre mice have enhanced antigen driven expansion of CD8^+^ T cells *in vivo* compared to their wild type counterparts in response to a tumor vaccine. Next, we wanted to determine if the deletion of Spry1 in T cells led to more robust rejection of tumors. To this end Wt and Spry1^Flox/Flox^ Lck Cre mice were subcutaneously injected with EL-4 tumor cells. Mice were then monitored for tumor presence and size. By day 13, 100% of the Wt mice demonstrated palpable tumor (Figure 8A). Alternatively, only 40% of the Spry1^Flox/Flox^ Lck Cre mice developed tumor. Likewise, the size of the tumors that did develop in the Spry1^Flox/Flox^ Lck Cre mice were significantly smaller than those in the Wt mice (Figure 8B).

Since Spry1^Flox/Flox^ Lck Cre T cells were more efficient in rejecting EL-4 lymphoma cells. We wanted to determine if such was the case for another tumor model. Wt and Spry1^Flox/Flox^ Lck Cre mice were injected IV with 250,000 B16 melanoma cells. On day 14 post injection the mice were sacrificed and assessed for tumor lung nodules. Similar to what was observed with the lymphoma challenged mice, Spry1^Flox/Flox^ Lck Cre mice had significantly less lung nodules than the wt controls (Figure 8C). Overall, our data demonstrate the ability of Spry1^Flox/Flox^ Lck Cre T cells to not only respond robustly to a tumor vaccine (Figure 7), but also to mediate rejection of tumors in both a lymphoma and solid tumor model Figure 8).

## Discussion

TCR-induced NF-AT activation plays a central role in regulating both T cell activation and inhibition [1]. As such, the balance of NF-AT induced gene expression impacts the ultimate outcome of TCR recognition. We have previously proposed that the NF-AT dependent genes Egr-2 and Egr-3 play an essential role in NF-AT-induced inhibition of T cells [2], [8]. As transcription factors we have demonstrated the ability of Egr-2 and Egr-3 to both promote the expression of inhibitory molecules as well as inhibit the transcription of activating genes. In this report we identify Spry1 as an Egr-3 target gene and define its role in negatively regulating CD4^+^ and CD8^+^ T cell effector function. Specifically, Spry1 inhibits proximal TCR-induced signaling to down modulate both TCR-dependent NF-AT and AP-1 mediated activation. In CD4^+^ T cells this leads to diminished effector cytokine expression such as IL-2 and IFN-γ. In primary CD8^+^ T cells this leads to diminished cytokine production and cytotoxic capability. Indeed, deleting Spry1 led to enhanced anti-tumor activity.

Sprouty was originally described in the fly as an inhibitor of morphogenesis [11]. In mammals there are 4 known family members all of which are induced by RTK signaling and serve in general to then inhibit signaling. The potential role of Spry1 in regulating T cell activation was originally defined by Choi and colleagues [15]. By employing tat-mediated protein transfer, this group demonstrated the ability of Spry1 to inhibit T cell activation in T cell clones and previously activated T cells but actually enhance activation of naïve T cells. In a follow up study, by employing Jurkat T cells, this group demonstrated the ability of Spry1 to be recruited to the immunologic synapse upon TCR engagement [13]. Our findings in primary T cells support their observations. Mechanistically, they demonstrate that recruitment of Spry1 to the synapse leads to the inhibition of NF-AT and Erk signaling. Our data employing Spry1 overexpression constructs supports this observation and we extend these findings to demonstrate that Spry1^Flox/Flox^ Lck Cre T cells demonstrate increased T cell activation. Further, such findings are consistent with the work of Akbulut et al. who demonstrated that sprouty proteins inhibit receptor mediated phospholipase c activation [16]. Interestingly, the Choi group also demonstrated a decrease in NF-κB activation by overexpressing Spry1 whereas we did not [13]. The exact mechanism accounting for this difference is unclear. The ability to inhibit NF-AT and AP-1 mediated transcription can clearly be linked to the inhibition of PLC-γ-induced activation, how Spry1 might inhibit NF-κB remains to be determined.

Overall, our studies, along with the published literature suggest a model whereby upon T cell activation Spry1 expression is induced by Egr-3 and then recruited to the immunologic synapse to mediate a negative feedback loop by diminishing PLC-γ-induced downstream signaling. In Th1 cells, in the setting of TCR stimulation and the absence of costimulation (Signal 1 alone) we propose that Egr-3 inhbitory factors such as Cbl-b and Spry1 contribute to promoting T cell anergy. In the setting of full activation these proteins also serve to limit T cell activation. Indeed, cytokine production was higher in CD4^+^ T cells lacking Spry1 compared to WT controls. Likewise, Spry1^Flox/Flox^ Lck Cre CD8^+^ T cells demonstrated increased killing capability. Given the important role of Ca^++^ and MAP-kinase induced signaling in the immune system one might predict that Spry1 will also play an important role in regulating other components of the immune response such as B cells, macrophages and neutrophils. Interestingly, recent findings from our lab suggest that while Egr-3 plays an inhibitory role in Th1 T cell activation, this is not necessarily the case for other T cell subsets such as Th17 cells (Powell and Parkinson, unpublished findings). Likewise, it remains to be determined if Spry1 plays an inhibitory role in all T cell subsets. While the precise role of Spry1 in these systems has yet to be identified, another member of the sprouty family Sprouty-related Ena/VASP homology 1-domain-containing protein 1 (Spred1) has been shown to inhibit IL-3-induced MAP-kinase activation in hematopoietic cells [17]. Additionally, Spred1 has been shown to negatively regulate IL-5-induced eosnophilia in a mouse model of asthma [18]. Interestingly, sprouty2 is epigenetically silenced in a model of B cell lymphoma and its overexpression can inhibit proliferation in lymphoma cells [19].

We posit that the ability of Spry1 to mitigate T cell activation may play a role in preventing hyperactive immune responses leading to autoimmunity. Alternatively, Spry1 mediated negative regulation may hamper anti-tumor responses. To this end the Spry1^Flox/Flox^ Lck Cre mice demonstrate superior anti-tumor activity in both a mouse model of lymphoma and melanoma. In the lymphoma model we were able to demonstrate that the deletion of Spry1 in T cells led to a more robust response to a whole cell tumor vaccine. Such findings suggest that pharmacologically blocking Spry1 may prove to be a useful adjuvant for tumor (and potentially pathogen target) vaccines. Furthermore, inasmuch as tumor-induced T cell anergy inhibits the anti-tumor effector response, it is possible that blocking Spry1 during the effector phase of the anti-tumor response might further enhance the efficacy of tumor immunotherapy.

## Methods

### Mice and Cells

C57BL/6 Spry1 Flox mice were obtained from J. Licht (Mount Sinai School of Medicine, New York, New York^18^. C57BL/6 Lck-cre transgenic mice were purchased from Taconic Farms (model 4197). Spry1 Flox mice were crossed to Lck cre transgenic mice to produce homozygotes. All animal protocols were approved by the Institutional Animal Care and Use Committee of Johns Hopkins University. GM-CSF secreting B16 cells were a gift from C. Drake (Johns Hopkins University, Baltimore, MD)^19^. EL4 cells were purchased from ATCC (Manassas, Virginia). All splenocytes were expanded with 1ug/mL anti-CD3 for two days, followed by five days with recombinant IL-2 (1ng/mL).

### Antibodies and Reagents

Anti-CD3 (clone 2C11) and Anti-CD28 (clone 37.51) were purchased from BD PharMingen. Recombinant IL-2 was purchased from peprotech. Ionomycin was purchased from Sigma. Anti-Phospho-Plcγ1, Anti-Phospho-LAT, Anti-Phospho-ERK, and anti-actin antibodies were purchased from Cell Signaling Technologies. All flow cytometry antibodies and reagents were purchased from BD biosciences.

### Plasmids and Vectors

PCB6^+^ Spry1 vector was obtained from J. Licht (Mount Sinai School of Medicine, New York, New York) ^20^. Lenti-viral vectors containing Spry1 were generated by PCR cloning of Spry1 into the cFUGW lenti viral construct (a gift from L. Chang, Johns Hopkins University, Baltimore, MD). Lenti-viral DC104 Spry1 was generated by cloning a truncated Spry1 which lacks the final 104 c terminal amino acids into the cFUGW lenti-viral construct. The IL-2-luciferase construct was a gift from Dr. J. Ragheb (National Institutes of Health, Bethesda, MD).

### ChiP Assay

Chip assay was performed according to manufacturers protocol (Pierce).

### Luciferase Assays

Jurkat cells (12×10^6^) were incubated with 9 ug lentivirus plasmids plus 3 ug luciferase reporter plasmid and were electroporated at 300 V and 825 uF resistance with an Equibio electroporator (Bio-Rad), then were incubated for 24 h; transfection efficiency was assessed by flow cytometry. Equivalent numbers of GFP^+^ Jurkat cells were added to 96-well plates and were stimulated for 20 h with 1ug/ml of anti-CD3. Luciferase activity in the total cell lysate was measured with the Dual-luciferase reporter assay system (Promega).

### ELISA

IL-2 ELISA was performed using the mouse IL-2 ELISA kit (ebioscience) according to manufacturer’s guidelines.

### Western Blot

Western blots were performed as previously described ^2^.

### IP3 Measurements

IP3 measurements were performed using the Inositol-1,4,5-Trisphosphate [H-3] Radioreceptor Assay Kit (Perkin Elmer Cat# NEK064) according to manufacturer’s guidelines.

### Flow Cytometry

Previously activated cells were stimulated for 48 hours with Anti-CD3 and Anti-CD28. For intracellular cytokine staining Golgi stop (BD biosciences) was added for the final six hours of stimulation. All flow cytometry was performed on a BD FacsCaliber.

### In Vivo CTL Assay

Wild type C57BL/6 and Spry1^flox/flox^ Lck cre C57BL/6 mice were given an I.P injection of recombinant vaccinia virus expressing ovalbumin (1×10^6^ pfu). One week later wild type C57BL/6 splenocytes were stained with either a high or low concentration of CFSE (invitrogen Cat # C34554) and CFSE high cells were pulsed with ovalbumin peptide (3ug/mL). CFSE high and CFSE low cells were mixed 1∶1 and then I.V. injected into wild type and Spry1 Flox LCK cre mice. Sixteen hours later mice were sacrificed, spleens were removed and flow cytometry was performed to determine the ratio of CFSE high and CFSE low cells.

### Antigen-driven CD8^+^T cell Assay

Wild type and Spry1^flox/flox^ Lck cre mice were subcutaneously injected with a 1∶1 mixture of irradiated ovalbumin expressing EL-4 cells and irradiated GM-CSF secreting B16 cells. Seven days later mice were sacrificed and inguinal lymph nodes were isolated. Single cells suspensions were stained for CD8, CD44 and OVA specific MHC class I tetramer (Beckman Coulter #T03000). Flow cytometry was performed by gating on CD8^+^ CD44^+^ cells.

### EL4 Tumor Model

Wild type C57BL/6 and C57BL/6 Spry1^flox/flox^ Lck cre recombinase mice were injected subcutaneously with 1×10^6^ EL4 cells. Tumor progression was monitored as well as tumor volume determined by measurement with a caliper.

### B16 Melanoma Tumor Model

Wild type and Spry1 flox LCK cre recombinase mice were injected intravenously with 2.5×10^5^ B16 tumor cells. 14 days later mice were sacrificed and lungs were harvested. Lung tumor mets were counted.

### Statistical Analysis

A minimum of three and up to six replicates was done for all experiments presented. Data are presented as means and standard deviations. Comparisons within groups were done with a t test with repeated-measures. Differences were considered statistically significant at the p<0.05 level.

## Supporting Information

Figure S1 **Spry1 is recruited to the cSMAC.** 5C.C7 T cells were incubated with peptide for 20 minutes, permeabilized and stained using anti-Talin and anti-Spry1 antibodies. Upper left shows a T cell-APC conjugate. Talin (green) is shown to delineate the pSMAC. Spry1 (red) mobilizes to the cSMAC region. Note, Spry1 is diffusely expressed in the APC. In the bottom panel the image is rotated to show the contact view. The green defines the pSMAC while the red clearly demonstrates Spry1 in the cSMAC region.
(TIF)

Figure S2 **Schematic of Wt and mutated Spry1 constructs.**
(TIF)

Figure S3 **Expression of Spry1 and mutated Spry1.** Western blot analysis of transfected Jurkat cells with either the empty vector, DC104 Spry1 (mutated) and full length Spry1. Note the antibody recognizes the endogenous Spry1 as well as a lower molecular weight form of the mutated construct.
(TIF)
