# Supplementary figures and images for: Regulation of CD4+ and CD8+ Effector Responses by Sprouty-1
Source: PLoS One. 2012 Nov 15;7(11):e49801. doi: 10.1371/journal.pone.0049801 (PMC3499516; doi:10.1371/journal.pone.0049801)

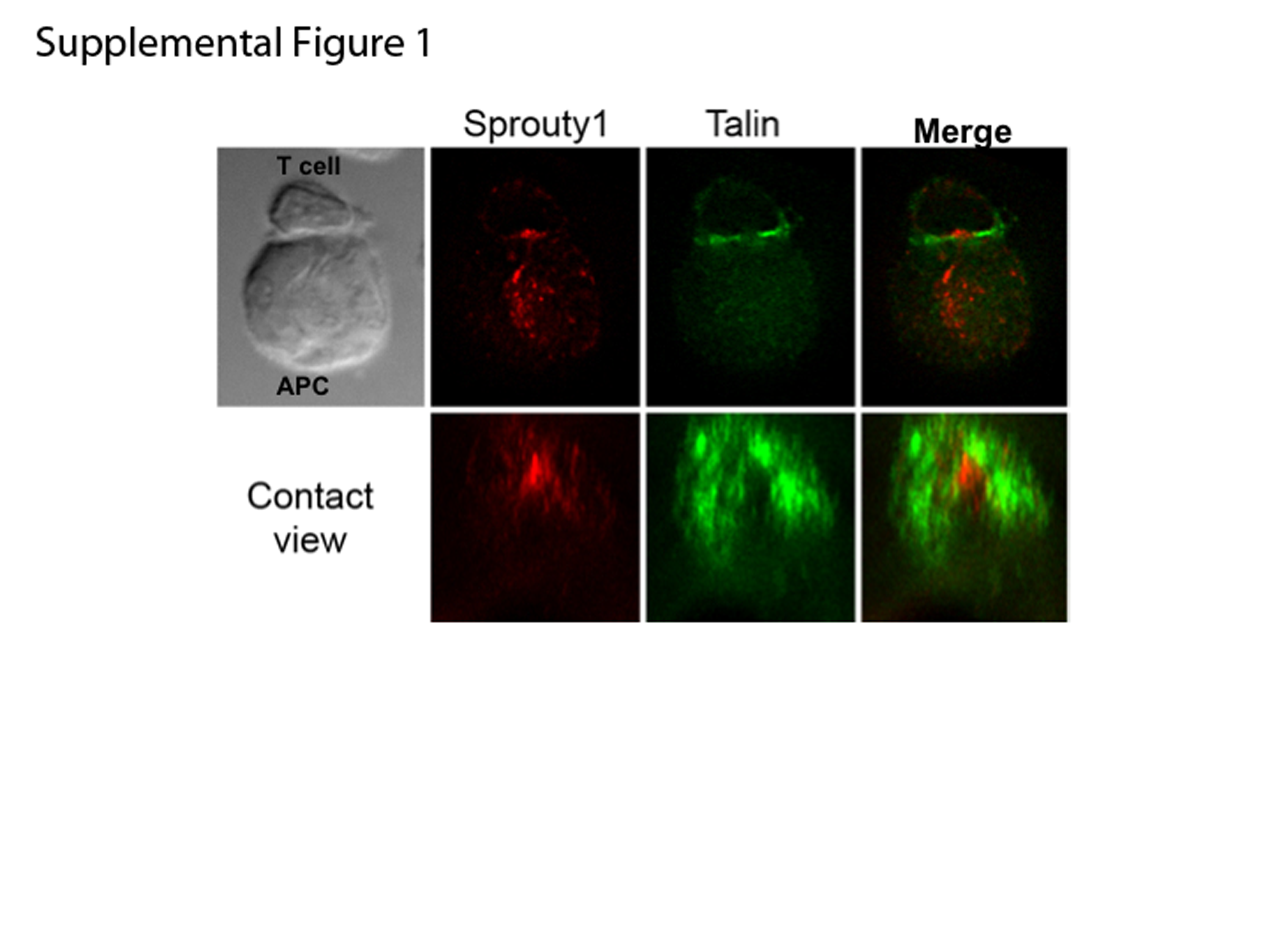

Supplement: Figure S1 — Spry1 is recruited to the cSMAC. 5C.C7 T cells were incubated with peptide for 20 minutes, permeabilized and stained using anti-Talin and anti-Spry1 antibodies. Upper left shows a T cell-APC conjugate. Talin (green) is shown to delineate the pSMAC. Spry1 (red) mobilizes to the cSMAC region. Note, Spry1 is diffusely expressed in the APC. In the bottom panel the image is rotated to show the contact view. The green defines the pSMAC while the red clearly demonstrates Spry1 in the cSMAC region. (TIF) [file pone.0049801.s001.tif]

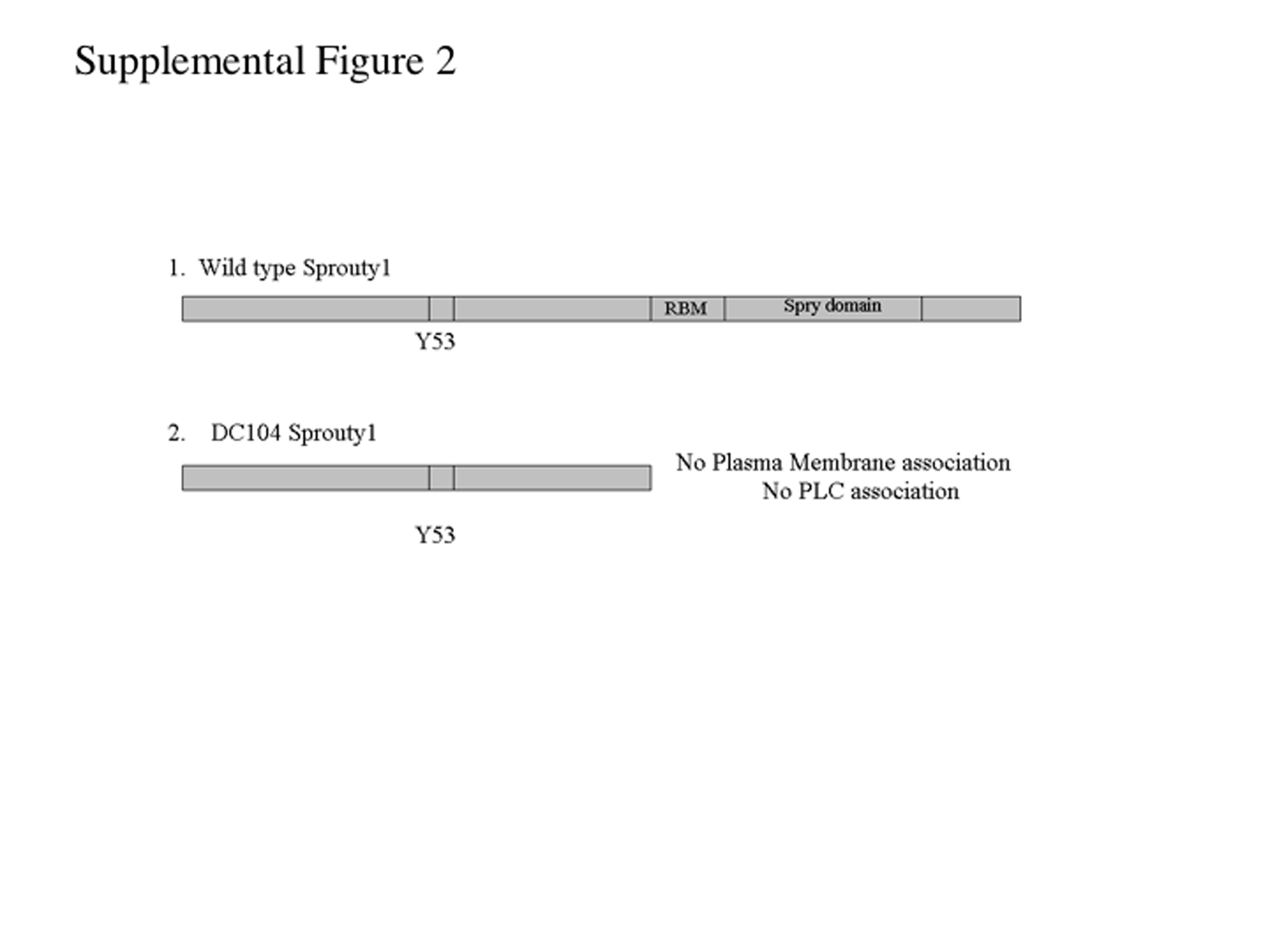

Supplement: Figure S2 — Schematic of Wt and mutated Spry1 constructs. (TIF) [file pone.0049801.s002.tif]

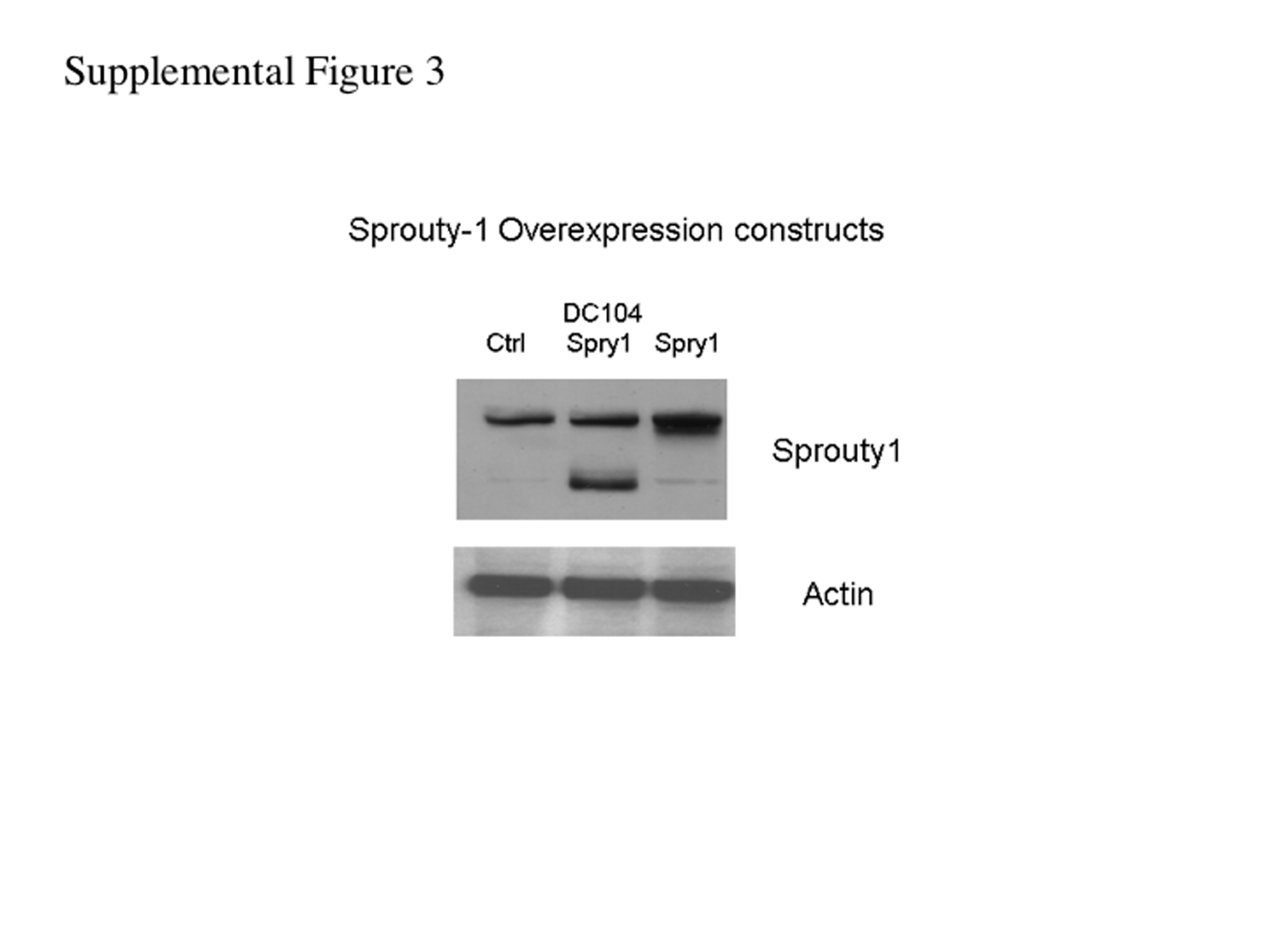

Supplement: Figure S3 — Expression of Spry1 and mutated Spry1. Western blot analysis of transfected Jurkat cells with either the empty vector, DC104 Spry1 (mutated) and full length Spry1. Note the antibody recognizes the endogenous Spry1 as well as a lower molecular weight form of the mutated construct. (TIF) [file pone.0049801.s003.tif]
